# Retinal granular cell tumor: a case report

**DOI:** 10.1186/s12886-021-02219-4

**Published:** 2021-12-27

**Authors:** Jimin Park, Kyung-Ja Cho, Junyeop Lee

**Affiliations:** 1 Department of Ophthalmology, Asan Medical Center, University of Ulsan College of Medicine, 88, Olympic-ro 43-gil, Songpa-gu, Seoul, 05505 Republic of Korea; 2 Department of Pathology, Asan Medical Center, University of Ulsan College of Medicine, 88, Olympic-ro 43-gil, Songpa-gu, Seoul, 05505, Republic of Korea

**Keywords:** Granular cell tumor, Intraocular tumor, Case report

## Abstract

**Background:**

To report a rare case of granular cell tumor invading the retina.

**Case presentation:**

A 56-year-old female complained of blurred vision for 2 weeks in her left eye. An irregular-shaped retinal mass in the inferonasal and extending to the optic disc accompanied by dense exudation and extensive serous retinal detachment was observed. Several intravitreal bevacizumab injections were ineffective for stabilizing retinal exudation and intraocular pressure (IOP). Vitrectomy was performed to re-attach the retina and obtain a tumor biopsy specimen. Histopathological analysis revealed that the intraocular mass was a granular cell tumor. Immunohistochemical studies demonstrated that the tumor was positive for S100 and CD68, focal positive for neurofilaments, but negative for ERG and HMB-45. Local recurrence and distant metastasis were not found, but visual acuity had worsened to no light perception at the last visit due to uncontrolled intraocular pressure and retinal exudation after the surgery.

**Conclusions:**

Granular cell tumor is a rare benign neoplasm, but it can lead to devastating visual loss if it invades the retina adjacent to the optic nerve head.

## Background

Granular cell tumor (GCT), also known as Abrikossoff tumor, is a rare soft-tissue neoplasm thought to originate from Schwann cells [1]. GCTs are mostly benign and often affect the head and neck; only 3% of GCTs have been observed in the orbit [2]. Furthermore, GCTs are extremely rare inside the eye, including the retina and choroid. Here, we describe a rare case of a retinal mass surgically excised and histologically diagnosed as a GCT.

## Case presentation

**Fig. 1:**
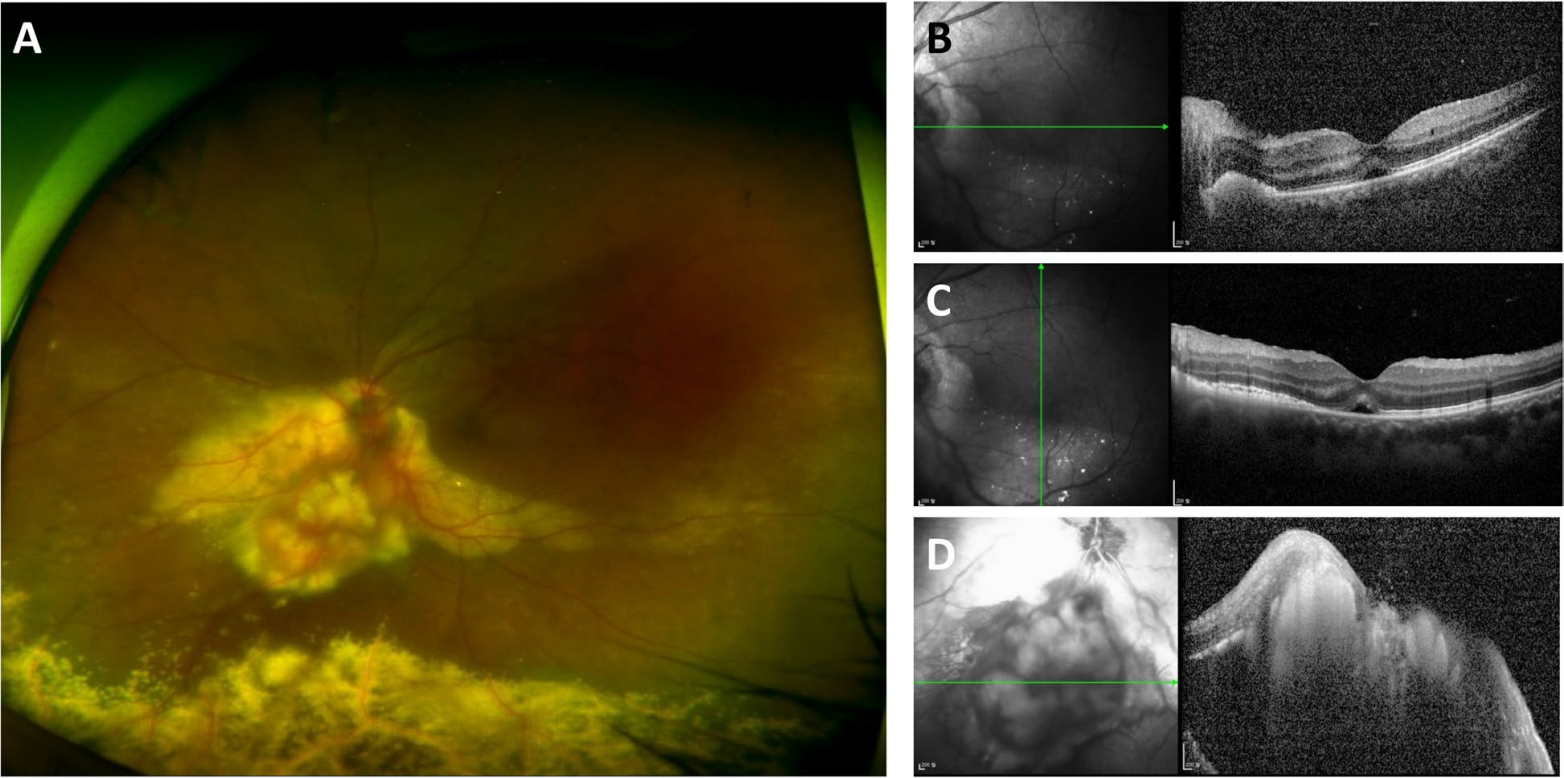
Fundus photography and optical coherence tomography (OCT) of the patient’s left eye. **A** Widefield fundus photograph showing an irregular-shaped retinal mass located at the inferonasal to optic nerve and hard exudate around the mass. Inferior exudative retinal detachment is also shown. **B**, **C** Subretinal fluid and photoreceptor degeneration are shown in the OCT scans. **D** A hyperreflective mass invading the retina and adjacent subretinal fluid can be detected

A 56-year-old woman complaining of blurred vision in her left eye for 2 weeks visited our clinic. At the first presentation, the best corrected visual acuity was 20/30 in the left eye. She didn’t have any underlying systemic disease. A fundus examination (Fig. 1A) showed a solitary, irregular-shaped, approximately 10 optic disc-sized yellowish to white mass located within the inferonasal and extending to the optic nerve head. Hard exudates around the mass lesion and exudative retinal detachment were identified.

**Fig. 2:**
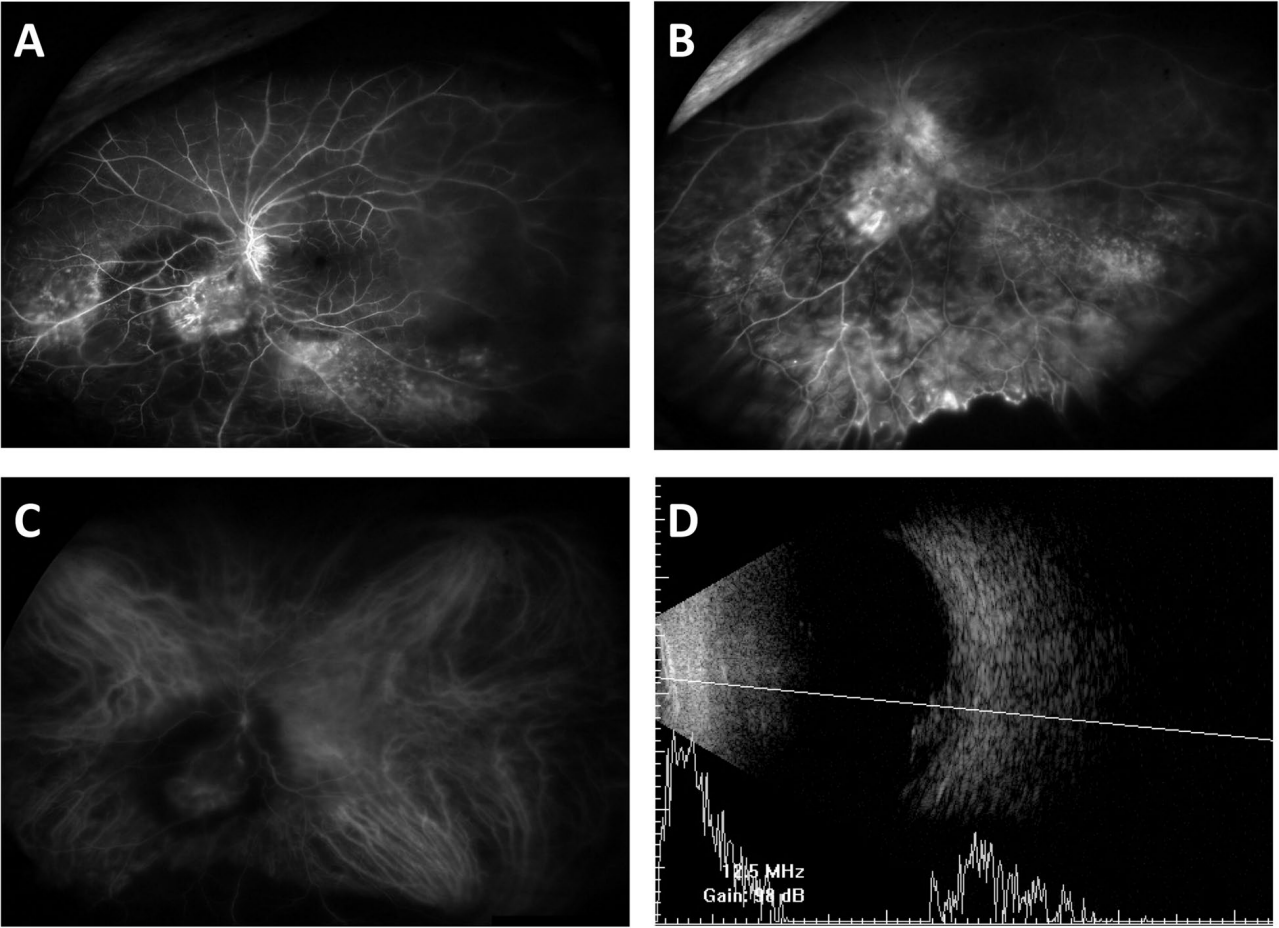
Fluorescein angiography (FA), indocyanine green angiography (ICGA), and B-scan ultrasonography of the patient’s left eye. **A**, **B** High vascularity of the mass shown by FA. Disc leaking is shown in the early phase, and capillary telangiectasis and a nonperfusion area can be seen. **C** The mass lesion has high vascularity, and ICGA shows a ring-shaped hypocyanescent halo around the mass is shown around the mass. **D** Homogeneous discoid mass with medium to high internal reflectivity shown by B-scan ultrasonography. There is no acoustic hollowing, uveal excavation, or extraocular extension

**Fig. 3:**
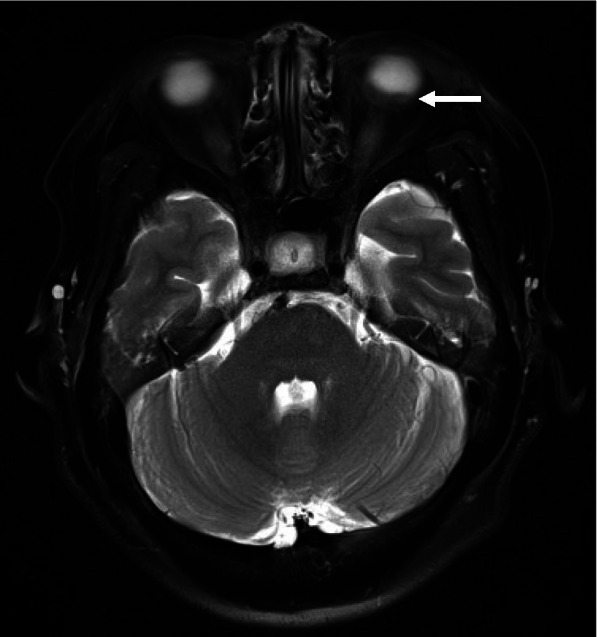
Magnetic resonance imaging (MRI) T2-weighted horizontal scan of the orbit. Approximately 1.7-cm-lesion with intermediate signal intensity in the left eyeball on T2-weighted image (white arrow). No definite lesion is observed in the optic nerve and brain parenchyma

Multiple bevacizumab injections were performed at another clinic to treat suspected Coat’s disease. Optical coherence tomography (OCT) (Fig. 1B, C, D) revealed an irregular margin of a hyperreflective retinal mass invading the retinal tissue. A small amount of subfoveal fluid and diffuse photoreceptor damage suggesting repetitive retinal detachment were found in OCT scans. A highly vascularized retinal mass and intratumoral vascular leakage were noted in the fluorescein angiography (FA) (Fig. 2A, B). In addition, early disc leakage, capillary telangiectasia at the detached retina, and a nonperfusion area at the peripheral retina were found in the late-phase FA. In the indocyanine green angiography (ICGA), a ring-shaped hypocyanescent halo around the mass due to hard exudates was observed (Fig. 2C). A definite direct connection to the other orbital tissues was not observed. A homogeneous discoid mass showing medium to high internal reflectivity without calcification was examined by B-scan ultrasonography (Fig. 2D). Brain magnetic resonance imaging (MRI) showed an approximate 1.7-cm-lesion with intermediate signal intensity on T2-weighted images and high signal intensity on T1-weighted images in the left eyeball (Fig. 3).

**Fig. 4:**
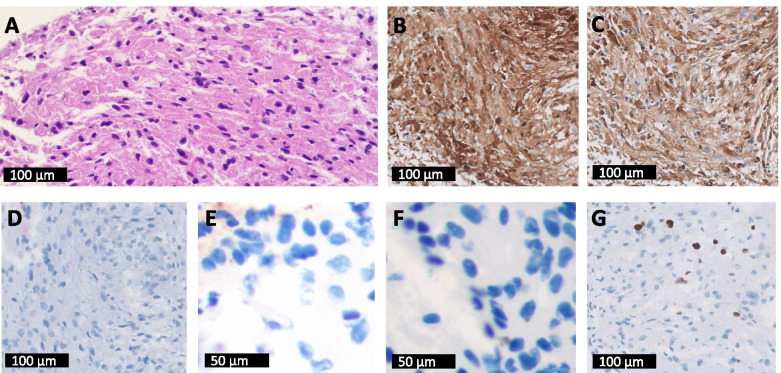
Immunohistochemical analysis of the excised tumor. **A** Hematoxylin and eosin staining: The lesion is microscopically composed of large polygonal cells with abundant eosinophilic granular cytoplasm and small round to oval nuclei. **B** S100: positive **C** CD68: positive; **D** HMB-45: negative; **E** neurofilament (cytology): focal positive; **F** ERG (cytology): negative; **G** Ki-67: 5%. (**A**, **B**, **C**, **D**, **G**: Philips digital pathology scanner; original magnification × 200, **E**, **F**: microscope; original magnification × 400)

No primary tumorous condition or distant metastasis was found during the systemic work-up. A vasoproliferative tumor was suspected, so off-label intravitreal bevacizumab injections were given, which were transiently effective only for suppression of exudation and did not reduce the mass lesion. Vitrectomy combined with retinal excisional biopsy and silicone oil tamponade was performed to provide an accurate histological diagnosis and reattachment of the retinal detachment. The histopathological analysis examination finally confirmed the intraocular mass as a GCT. The tumor was composed of large polygonal cells with abundant eosinophilic granular cytoplasm and round nuclei (Fig. 4A). To differentiate the primary tumor from other possibilities, such as vasoproliferative tumors and choroidal melanomas, we performed additional immunostaining. In the immunohistochemical studies, the tumor was positive for S100 and CD68 but focal positive for neurofilament and negative for ERG and HMB-45 (Fig. 4). The percentage of Ki-67 was 5%, which excluded the possibility of malignant primary tumors (Fig. 4G).

We tried to remove the tumor as much as possible, but a part of the tumor was left behind to minimize the damage of the normal retina and choroidal tissues. There was no local recurrence or metastasis up to the last visit 6 months after surgery. However, high intraocular pressure was uncontrolled after surgery under the silicone oil tamponade and recurrent subretinal fluid originated from the remained tumor. The visual acuity of the left eye worsened into no light perception.

## Discussion and conclusion

First mislabeled as ‘granular cell myoblastoma’, GCTs were previously thought to have originated from striated muscle. However, a previous study reported that there was no similarity between GCTs and striated muscle under electron microscopy [3], and GCTs have been reported in several organs other than striated muscle [4]. Recently, the origin of GCTs has been identified as neural lesions because of the similarity of cytoplasmic granules between GCTs and Schwann cells [1], which is still a matter of debate.

Orbital GCTs are known to be about 3% of GCTs [2]. Most patients complain of extraocular muscles (EOM) limitation, diplopia, and proptosis because orbital GCTs mainly affect EOMs, especially the inferior rectus muscle [5, 6]. This association between GCTs and EOMs can be explained by the dense neural supply in EOMs.

The prevalence of intraocular GCTs has been very low since the first case was reported in 2019 [7]. The occurrence of GCTs in the eyeball might be correlated with the optic nerve. About 30% of orbital GCTs affect the optic nerve due to tumor adhesion to the optic nerve, which disturbs visual acuity [8]. Although the optic nerve originates from the central nervous system, it does not consist of Schwann cells. Nevertheless, recent studies have described GCTs as a neural Schwann cell-related neoplasm [1, 4]. This discordance on the origin of GCTs might be partially explained by the hypothesis that GCTs are biologically heterogeneous tumors [9]. The poor visual outcome and retinal mass adjacent to the optic nerve head in our case cannot completely exclude the possibility that the retinal mass had originated from the optic nerve or resulted from microscopic invasion of the retinal mass into the optic nerve, although unproven in the imaging studies. In this study, it was hard to differentiate whether the origin of the tumor is the choroid or retina by histopathological examination.

Considering the very low incidence of intraocular GCT, it was difficult to diagnose this case as a GCT without biopsy, and differential diagnosis was needed because we had misdiagnosed it as a vasoproliferative tumor. GCTs are usually diagnosed by histopathological examination and characterized by the presence of sheets of large, polygonal, elongated cells with numerous eosinophilic granular cytoplasms [10]. Most GCTs express S100 protein, vimentin, CD68, NK1-C3, and neuron-specific enolase (NSE). Glial fibrillary acidic protein (GFAP), neurofilament marker, and chromogranin-A are negative in GCTs. These immunohistochemical findings of GCTs were exactly consistent with our case.

To differentiate from other tumors, additional immunohistochemical studies and examinations were performed. Accumulation of secondary lysosomes in the cytoplasm presents as abundant granules, which is positive for CD68 [11]. This nonspecific change can be observed in many other tumors and is derived from non-neural cells, such as connective tissue, smooth muscle, epithelial, and endothelial cells [12]. HMB-45 and Melan-A are positive in melanoma, which also expresses strong S100. Moreover mushroom-shaped mass, acoustic hollowing, and choroidal excavation are the typical findings of choroidal melanoma in B-scan ultrasonography [13]. Vasoproliferative tumors, which might be confused with GCTs because of a discoid mass with medium to high internal reflectivity on B-scan ultrasonography, show a mix of vascular and glial proliferation in microscopic studies [14]. Malignant GCTs are even rarer, and about 1–2% of GCTs are malignant [8]. The Ki-67 index value in this case was 5%, which indicated a benign tumor.

We believe that this is the first case report to describe an intraocular GCT diagnosed in an adult. Since intraocular GCT is very rare, we compared our adult case with a pediatric intraocular GCT case [7]. Both intraocular masses were found in the retina adjacent to the optic nerve without involvement of extraocular muscles or other orbital tissues. Both patients showed a highly vascularized mass on FA. Although the pediatric patient showed no obvious leakage in FA [7], our adult patient showed intratumoral vascular leakage in FA. Therefore, OCT and widefield ICGA were helpful to determine the tumor extent in our case, which was not evaluated in the previous case. However, it was difficult to determine choroidal invasion due to irregular margin of the tumor shown in OCT and B-scan ultrasonography. Because overall, GCTs occur most commonly in the fourth to sixth decade [10], our case is more appropriate in the context of age when tumors are prevalent. Therefore, it is essential to differentiate an intraocular GCT from other primary intraocular tumors, including melanomas and vasoproliferative tumors.

There is no guideline for GCT treatment, but complete resection has been recommended for treatment of intraorbital GCT [8]. Chemotherapy and radiotherapy can be considered for additional treatment in malignant GCTs, but their effectiveness has not been convincingly demonstrated [15]. Pazopanib is a novel strategy for treating a malignant GCT [16]. Still, complete surgical excision is suggested to assure no recurrence. Less than 2% of GCTs are malignant, but these are aggressive and associated with a poor prognosis; therefore, regular workup is needed for local recurrence and distant metastasis surveillance.

Devastating visual loss is possible when GCTs occur in the eyeball. These tumors are very rare and usually benign. Since it is difficult to diagnose a GCT without biopsy, ophthalmologists should be aware of these tumors when they encounter a patient presenting with an intraocular mass.

## Data Availability

The data and materials are presented within the manuscript.

## Abbreviations

GCT: Granular cell tumor
OCT: Optical coherence tomography
FA: Fluorescein angiography
EOM: Extraocular muscle
MRI: Magnetic resonance imaging
ICGA: Indocyanine green angiography
IOP: Intraocular pressure
